# Advances in Enzyme and Ionic Liquid Immobilization for Enhanced in MOFs for Biodiesel Production

**DOI:** 10.3390/molecules26123512

**Published:** 2021-06-09

**Authors:** Reem Shomal, Babatunde Ogubadejo, Toyin Shittu, Eyas Mahmoud, Wei Du, Sulaiman Al-Zuhair

**Affiliations:** 1 Chemical and Petroleum Engineering Department, UAE University, Al Ain 15551, United Arab Emirates; 201350343@uaeu.ac.ae (R.S.); 201990035@uaeu.ac.ae (B.O.); 201990179@uaeu.ac.ae (T.S.); emahmoud@uaeu.ac.ae (E.M.); 2 Department of Chemical Engineering, Tsinghua University, Beijing 100084, China; duwei@tsinghua.edu.cn; 3 National Water and Energy Center, UAE University, Al Ain 15551, United Arab Emirates

**Keywords:** biodiesel, metal–organic frameworks, immobilization, lipases, ionic liquids

## Abstract

Biodiesel is a promising candidate for sustainable and renewable energy and extensive research is being conducted worldwide to optimize its production process. The employed catalyst is an important parameter in biodiesel production. Metal–organic frameworks (MOFs), which are a set of highly porous materials comprising coordinated bonds between metals and organic ligands, have recently been proposed as catalysts. MOFs exhibit high tunability, possess high crystallinity and surface area, and their order can vary from the atomic to the microscale level. However, their catalytic sites are confined inside their porous structure, limiting their accessibility for biodiesel production. Modification of MOF structure by immobilizing enzymes or ionic liquids (ILs) could be a solution to this challenge and can lead to better performance and provide catalytic systems with higher activities. This review compiles the recent advances in catalytic transesterification for biodiesel production using enzymes or ILs. The available literature clearly indicates that MOFs are the most suitable immobilization supports, leading to higher biodiesel production without affecting the catalytic activity while increasing the catalyst stability and reusability in several cycles.

## 1. Introduction

The need to protect the environment from fossil fuel emissions together with the continuously growing energy needs has led to focus on renewable energy sources [1,2,3,4,5,6,7,8,9]. Apart from their expected depletion in the future, fossil fuels have unstable prices, intensifying the search for more sustainable and reliable energy sources [10]. Ideal fuel substitutes should possess better properties than conventional fuels, such as renewability, nontoxicity, biodegradability, and less-than-zero release of harmful gases into the environment [11,12,13]. Possible alternative energy sources include sunlight, wind, and biofuels [14].

Biodiesel is gaining increasing recognition worldwide due to the abundance of various possible feedstocks [15,16,17,18,19,20] and its superior properties compared to petroleum diesel, including better cetane number, higher flash point, and zero sulfur content [21]. These benefits along with its almost direct use in the diesel engine have encouraged the replacement of petroleum diesel with biodiesel [7,10,21,22,23,24,25,26]. Biodiesel is mainly produced by the transesterification of triglycerides and the esterification of free fatty acids (FFA) found in vegetable oils and animal fats [27]. However, in biodiesel production, the feedstocks should be carefully selected and the production process should be optimized for economic competitiveness with petroleum diesel production. For instance, use of waste oil instead of pure vegetable oil can effectively reduce production costs. However, such feedstocks suffer from inconsistent availability and collection complexity [14,28,29,30,31,32,33]. Microalgae appear to be the most promising feedstock for biodiesel production, as they can generate high lipid amounts, their cultivation does not require arable land, and many strains can grow in saline water.

## 2. Biodiesel Production

### 2.1. Conventional Catalysts

In addition to the appropriate feedstock selection, the catalyst used to convert oils into biodiesel plays an important role in the economic feasibility and high yield of the overall process [14,34,35]. Homogeneous chemical catalysts, especially alkaline catalysts, are commonly used in biodiesel production due to their fast reaction rates and high yield. However, these catalysts are corrosive, cannot be easily recycled, and should be washed out from the product, generating large amounts of wastewater [36,37,38,39]. Moreover, in the case of alkaline catalysts, the feedstock should be pretreated if its FFA content exceeds 1% to prevent saponification reactions, which consume the catalyst, reduce the yield, and complicate the downstream separation of the product [37]. In contrast, acid catalysts are not sensitive to FFA and can therefore be used to convert low quality feedstock into biodiesel without pretreatment. For instance, tin tetrachloride (SnCl_4_) can catalyze the esterification of *Zanthoxylum bungeanum* seed oil with >96% yield under optimum reaction conditions. However, compared to alkaline catalysts, acid catalysts have lower reaction rates and require higher alcohol/oil molar ratios to promote the reaction [40]. For example, sunflower oil transesterification was achieved in 91.7% yield upon treatment with sodium hydroxide for 1 h [41], whereas *Z. bungeanum* seed oil transesterification afforded 94% yield upon treatment with sulfuric acid for 12 h. Therefore, a two-step process has been developed to optimize the reaction yield. In particular, the feedstock was first esterified using an acid catalyst to reduce the FFA content in the oil, and then, the triglycerides were transesterified using an alkaline catalyst. Transesterification of *Spirogyra* sp. microalgae oil after 180 min at 40 °C using a mixture of potassium hydroxide, sodium hydroxide, and sulfuric acid as the catalysts at an oil/methanol ratio of 1:3 and a catalyst loading of 1.5% resulted in a biodiesel yield of 96.9%. In contrast, the application of KOH alone under the same reaction conditions afforded a biodiesel yield of 94.9% [42].

To overcome the drawbacks of homogeneous catalysts, heterogeneous solid catalysts have been used in many organic reactions [43], as they can reduce the soap formation and can be easily separated and reused. Moreover, heterogeneous catalysts can be directly employed in continuous flow reactors [34,44]. However, their application is restricted by their mass transfer limitations and low thermal stability [14].

Recently, biocatalysts and ionic liquids (ILs) have attracted increasing attention as alternatives to conventional chemical catalysts [45,46,47]. Pristine and functionalized metal–organic frameworks (MOFs) have also been shown to improve the performance of heterogeneous catalysis due to their high-order structure, high porosity with high specific surface area, and tunable acidity [48]. In this review, we explore the recent advances in catalytic biodiesel production with particular emphasis on enzymatic reactions and innovative ways of immobilizing enzymes and ILs on MOFs.

### 2.2. Enzymatic Biodiesel Production

Lipases are used as alternatives to chemical catalysts in biodiesel production. Since the lipases catalyze the esterification of FFA, the reaction conditions are mild and the saponification reaction is prevented [49]. However, the enzymatic reactions are relatively slower when using lipases than alkaline catalysts. Furthermore, the enzymes may be inhibited by methanol, the most commonly used reactant in enzymatic biodiesel production. Using the enzymes in soluble form hinders their separation and reuse [50]. Therefore, enzymes are immobilized on porous supports with high surface area to enhance their stability and facilitate their separation and reuse. Nevertheless, immobilized enzymes suffer from mass transfer limitations and glycerol deposition as a byproduct in the pores of the support, preventing the substrate from reaching the enzyme active cites.

#### 2.2.1. Factors Affecting Enzymatic Biodiesel Production

##### Lipid Source

Biodiesel can be produced from crop oils [51,52,53], waste cooking oils [54,55,56,57,58,59], animal fat [60,61,62,63,64,65,66], and microalgae oils. Hence, earlier studies have focused on determining the chemical composition of several lipids in these raw materials [67]. The lipids used in biodiesel production differ in their agronomic characteristics, and the content of FFA, water, and phospholipids have the greatest effect on biodiesel quality. Compared to alkali-catalyzed transesterification, the FFA content in the biodiesel feedstock does not affect the enzymatic reaction, as lipases can directly esterify FFA to produce biodiesel. However, phospholipid concentrations of >1% in raw oils can stop the generation of biodiesel, as reported for the transesterification catalyzed by *Candida antarctica* lipase [68].

In the case of heterogeneous catalysts, whether chemical catalysts or immobilized enzymes, the effects of water and FFA content on transesterification are less significant. However, water inhibits the acid-catalyzed transesterification of raw oils. It has been shown that the conversion of soybean oil to methyl esters decreased from 90.5% to 58.8% when the FFA content increased from 5% to 33%. In contrast, when H_2_SO_4_ was used as the catalyst, a yield of 90% was only afforded upon reaction with 3.0 wt % H_2_SO_4_ and a methanol/oil molar ratio of 6:1 at 60 °C for 96 h only when the water content in soybean oil was below 0.5 wt %.

Interestingly, water also affects the stability and catalytic activity of lipase-catalyzed transesterification in nonaqueous media. Lipases distinctly act at interfaces of organic and aqueous phases, and their activity depends on the interfacial area. A small amount of water is therefore required to maintain the enzymatic activity in organic solvents as it increases the available interfacial area. However, excess water promotes the hydrolysis of the oil. Thus, the optimum water content should be carefully identified to maximize the enzymatic activity [69].

Lipases exhibit interfacial activation, which is an increase in activity when the substrate (lipid) forms a distinct phase near the adsorbed enzyme. X-ray analyses have shown that lipases contain an amphiphilic amino acidic chain, known as the lid, which is mobile and protects the enzyme active sites, and thus, it is responsible for the enzyme activation [70]. When the lid is closed, the active sites are protected from the environment and the lipase remains inactive. Lipase activity is observed only in the open conformation.

##### Alcohols

Methanol is the most commonly used alcohol in enzymatic biodiesel production and is added in excess to improve the reaction rate and yield. However, high alcohol concentrations cause unfavorable unfolding of the enzyme to a more helical state by stripping essential water molecules, thus impairing its activity [71]. In addition, high alcohol/triglyceride ratios increase glycerol solubility and affect its separation.

Several measures have been proposed to overcome the inhibition of lipases by methanol, including the stepwise addition of methanol, the use of another acyl acceptor, and the introduction of a suitable solvent that dissolves methanol. For immobilized *Candida antarctica* lipase, the stepwise addition of methanol resulted in 98.4% conversion of vegetable oil in 48 h [72]. Similar results were observed for lipases from other sources, such as *Pseudomonas fluorescens* [73], *Rhizopus orzyae* [74], and *Candida* 99–125 [75]. Moreover, over 87% of the initial enzyme activity was maintained at the end of the process with the stepwise addition of methanol. However, this method is complex and cannot be applied to large-scale industrial sectors [76].

Methanol inhibition occurs when its amount in the reaction medium exceeds its solubility. Due to its low solubility in oils, methanol and oil separate at concentrations just above their stoichiometric ratio. Subsequently, the alcohol molecules strip off the essential water microlayer surrounding the lipase, which is required to maintain the conformation and catalytic activity of the enzyme [77]. Therefore, an organic solvent is commonly added to increase the solubility of the substrates and reduce the inhibitory effect of methanol [78]. The addition of an organic solvent can also reduce the viscosity of the reaction medium and enhance the stability and recovery of the immobilized enzyme [79]. Among the possible organic solvents, *n*-hexane is most commonly used in enzymatic transesterification reactions, as it can enhance lipase activity and biodiesel productivity. For example, the biodiesel yield using *Mucor miehei* lipase at a 3:1 methanol/oil ratio increased from only 19% in the solvent-free system to 95% using *n*-hexane within the same reaction time of 5 h [80]. However, most of the suitable organic solvents are toxic and volatile, leading to hazardous effects. Furthermore, an additional purification step is required to remove the second organic solvent from the final product, increasing the cost and energy demand of the process. In addition, the use of organic solvents with immobilized lipases causes the deposition and adsorption of glycerol due to its low solubility in hydrophobic solvents. Thus, an outer glycerol film is formed, which reduces the mass transfer of the hydrophobic substrates to the enzyme active sites, leading to lower reaction rates [71].

Replacing methanol with ethanol as the acyl acceptor can also reduce the inhibitory effect of methanol. A biodiesel yield of 91% was achieved in 90 min with a methanol/oil molar ratio of 10.44:1 using KOH as the catalyst at 66.8 °C. In contrast, the yield was reduced to 77.4% using an ethanol/oil molar ratio of 8.42:1 and KOH as the catalyst for 120 min at 61.3 °C. These results also indicated that the temperature plays a more significant role in methanolysis compared to ethanolysis. Moreover, the separation of ethyl esters from glycerol was more difficult compared to that of methyl esters [80].

#### 2.2.2. Immobilized Enzymes in Biodiesel Production

Immobilization is defined as the attachment of an enzyme onto an insoluble solid support material. In addition to the easy reuse in continuous reactors, immobilization endows lipases with shear and thermal stability as well as easy downstream processing [81]. Furthermore, their confinement in the porous structure of the support protects them from harsh media. Reusability is essential for the feasible application of high-cost enzymes and is the most practical approach for their industrial application. Enzyme immobilization methods can be classified as adsorption, covalent bonding, entrapment, and cross-linking, which together with the appropriate support material play a significant role in the development of an efficient lipase [82]. Moreover, enzyme immobilization greatly relies on the amine functional group of the amino acids in the enzyme, which contribute to the binding to the support.

Despite the advantages of immobilized enzymes, several drawbacks still exist that limit their use in biodiesel production, including the (1) loss of enzymatic activity during immobilization, (2) high cost of carriers, (3) low stability in oil–water systems, (4) large mass transfer limitation, and (5) glycerol adsorption [9]. To improve the properties of immobilized enzymes, coordinated matrices with mesoporous structure and average surface area, such as MOFs, should be used to facilitate substrate diffusion through the pores while reducing enzyme leaching [81].

## 3. MOFs

### 3.1. MOF Structure and Properties

As already discussed, heterogeneous catalysts suffer from mass transfer limitations and catalytic instability. Therefore, MOFs have been employed as support to improve the performance of heterogeneous catalysts [14,49,50,83] due to their high crystallinity, high porosity, and strong interactions in their metal–ligand network [84]. MOFs can be easily prepared with high surface area (5900 m^2^/g) and specific volume (2 cm^3^/g) [36,84,85,86,87,88,89,90,91,92,93,94,95,96,97,98]. Here, we review the recent advances in biodiesel production using modified catalysts. We focus on enzyme and IL immobilization on MOFs, the balance between increased stability and reusability of the immobilized enzyme and mass transfer limitations, pore size and porosity control, manipulation of the hydrophobicity/hydrophilicity in the reaction medium, and optimization of the biodiesel production process.

### 3.2. MOF Preparation

MOFs are porous polymers comprising metal-containing nodes and organic ligands linked through coordination bonds [82]. They possess unique characteristics, such as tunable ultrahigh porosity (up to 90% free volume), large surface area (>6000 m^2^/g), diverse functionality, high thermal and mechanical stability, and good electronic properties [99]. Owing to these properties and the wide variety of organic and inorganic components in their structures, MOFs have been widely studied in the fields of storage, separation, catalysis, biomedical applications, and sensor materials [90]. For example, MOFs have been effectively used for gas storage (e.g., H_2_, CH_4_, CO_2_, and NO) without the need for high pressure and/or compression as well as for the separation of toxic organic compounds [100]. Various cost-effective, green, and rapid synthetic methods have also been developed, which can be classified as solvothermal, slow evaporation/direct precipitation, microwave-assisted, electrochemical, mechanochemical, and sonochemical [101] (Table 1). These classifications are selected based on the type of metal, organic linker, and targeted application [99].

#### 3.2.1. Conventional Methods

In conventional solvothermal synthesis, a mixture of metal ions and organic linkers in an appropriate organic solvent is heated in a glass vial for low temperature processes or in a Teflon-lined autoclave or bomb reactor for temperatures > 400 K. If water is used as the solvent, the method is referred to as hydrothermal. The desired MOF structure can be prepared by controlling the reaction parameters, including pressure, temperature, solvent composition, and reagent concentration. When the reaction temperature is higher than the boiling point of the solvent, the reaction is referred to as solvothermal, while at reaction temperatures below the boiling point of solvent, the reaction is called nonisothermal. Moreover, some MOFs—such as MOF-5, MOF-74, MOF-177, HKUST-1, and ZIF-8—have been synthesized under ambient conditions [106]. For example, rod-like CuBTC-MOF particles were prepared via the solvothermal method using benzene-1,3,5-tricarboxylic acid (BTC) and divalent copper in 50 *v*/*v*% ethanol/water. CuBTC-MOF had a unit cell length of 37.12 nm, surface area of 1085.72 m^2^/g, and total pore volume of 1.68 cm^3^/g Utilization of 0.04 g CuBTC-MOF as the sole catalyst for biodiesel production from palm oil afforded a maximum yield of 91% after 4 h in a methanol/oil volume ratio of 5:1 at 60 °C [100].

#### 3.2.2. Microwave Synthesis

To improve the produced MOF’s crystallinity, a microwave-assisted method based on chemically inert metal ions has been developed to provide the energy required for the reaction and to homogeneously increase the temperature in a localized zone. Microwave synthesis is an environmentally friendly alternative to conventional heating, offering fast crystallization, narrow particle size distribution, and better morphological control of the target MOFs [101]. Cr-MIL-100 was the first MOF synthesized via the microwave-assisted method, achieving conversion yields of up to 97% [103].

#### 3.2.3. Sonochemical Synthesis

Sonochemical synthesis is a rapid and environmentally friendly method for the synthesis of MOFs using 10–20 MHz ultrasonic radiation in a homogeneous liquid. The cavitation created by the ultrasonic waves sharply increases the temperature and pressure, creating hot spots that facilitate the rapid formation of homogeneous MOF crystals [107]. Consequently, the crystallization time and particle size are significantly reduced compared to those of the conventional solvothermal synthesis. The first MOF synthesized by the sonochemical method was [Zn_3_(BTC)_2_] [108].

#### 3.2.4. Electrochemical Synthesis

For the electrochemical synthesis of MOFs, metal ions are continuously supplied through anodic dissolution instead of metals salts; they react with the linker molecules and a conducting salt dissolved in the reaction medium [108]. The method was first used for the preparation of HKUST-1 [Cu_3_(BTC)_2_], which afforded promising results in renewable fuel production [105].

### 3.3. Properties of MOF-Immobilized Enzymes

#### 3.3.1. Enzyme Stability

In general, enzymes are denatured at moderate temperatures and in the presence of chemical denaturants, but remain stable at very high pressures. Therefore, enzymes can be immobilized on a MOF structure to protect it from denaturation and to increase its stability. MOF–enzyme bioconjugates possess higher catalytic stability and thermal tolerance than free enzymes. For example, the half-life of a lipase encapsulated in zeolite imidazolate framework-8 (ZIF-8) at 55–75 °C was increased by 3.2 times and its deactivation rate decreased compared to that of the free enzyme [109]. The enhanced thermal stability of the ZIF-8-immobilized enzyme was attributed to the confinement of the enzyme inside the biocompatible microenvironment, which prevented protein unfolding. Additional studies have shown that the activity of enzymes immobilized on MOFs is not affected by exposure to denaturing organic solvents, such as methanol, ethanol, dimethylformamide, and dimethyl sulfoxide. The enzymes immobilized on MOFs retain 100% of their initial activity, in contrast to free enzymes, which retain only up to 20% of their initial activity under the same denaturation conditions [48].

#### 3.3.2. Enzyme Recovery and Reusability

An important advantage of enzyme immobilization is the efficient recovery and reuse of catalysts, which is particularly important for reducing the overall cost of enzyme-based procedures. For example, the residual activity of lipase@ZIF-8 after repeated use for seven cycles was 54% of that in the first cycle, while that after storage for 25 days was 90% of the initial activity [109]. A similar result was observed for laccase adsorbed on Zr-MOF, a bimodal micro-mesoporous MOF, where the residual activity was 50% of that in the first cycle after being used for 10 cycles. Furthermore, enzyme immobilization in MOFs reduces product contamination, thus affording lower impurities than free enzymes. However, the nano-size of the enzyme–MOF conjugates hinders their reusability on an industrial scale.

Recent studies have suggested the use of a new 3D matrix as a support for MOFs [110]. For example, a commercially available melamine sponge was tested as a 3D matrix for embedding *α*-amylase entrapped in ZIF-67 [111]. The melamine sponge was selected due to its low cost, low weight, and high nitrogen content, which provides enormous binding sites for enzyme–MOF bioconjugation [112,113]. The *α*-amylase embedded MOF–sponge matrix was synthesized by dip coating the melamine sponge in a solution of the pre-synthesized *α*-amylase entrapped in ZIF-67 at room temperature for 1 h. The *α*-amylase/ZIF-67 layers on the sponge skeletons were formed because of electrostatic and π–π stacking interactions [112].

#### 3.3.3. Allosteric Effect

Allostery involves the binding of a ligand, known as the effector, to the allosteric site of an enzyme, leading to conformational changes in the enzyme active site [114]. Effectors that enhance the enzymatic activity, such as oxygen and metal ions including Fe^3+^, Ca^2+^, and Zn^2+^, are also known as allosteric activators. For example, a CaHPO_4_–*α*-amylase hybrid biocatalytic nanosystem has been designed based on the allosteric effect using Ca^2+^ as the effector [115]. The immobilized *α*-amylase showed improved enzymatic activity in the hydrolysis of 2-chloro-4-nitrophenylmaltotrioside. Thus, the allosteric effect is very promising for improving biodiesel production using lipases immobilized on MOFs.

### 3.4. Lipase Immobilization on MOFs

**Figure 1:**
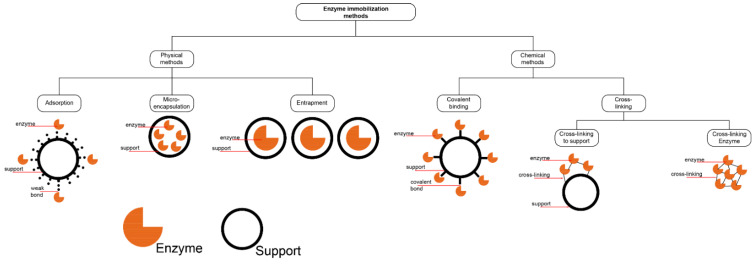
Enzyme immobilization methods.

An immobilized lipase can be defined as a lipase localized in a well-defined region without losing its activity, thus showing high reusability [116]. The main methods used to form immobilized lipase are physical (surface) adsorption, covalent binding, encapsulation, cross-linking, and in situ synthesis shown in Figure 1. The appropriate method for the preparation of each conjugate should be carefully selected, as it can significantly affect the enzymatic activity in the reaction.

#### 3.4.1. Physical Adsorption

In physical adsorption, enzymes are immobilized on a support matrix by weak interactions, van der Waals forces, hydrogen bonding, and electrostatic interactions. The main advantage of physical adsorption is that it does not affect the enzyme activity as the weak attraction forces do not alter its native structure and active sites [81]. MOFs can be used as an adsorption support matrix as they offer a large enzyme loading capacity due to their high surface area. In addition, the attachment of the enzymes on the surface of an already prepared MOF protects them from the harsh conditions applied to synthesize MOFs. Moreover, no functional groups are required in physical immobilization.

In a recent study, *Burkholderia cepacia* lipase that was physically immobilized on hierarchical zeolite imidazolate framework (BCL-ZIF-8) was tested for biodiesel production [81]. The immobilization efficiency depended on the adsorption time, immobilization temperature, pH, and morphology of ZIF-8. The biodiesel yield was 93.4% at a lipase loading of 700 mg and the activity recovery reached 98.8%. Interestingly, unlike other MOFs, ZIFs can be prepared at room temperature, but their pore size is very small (~1.5 nm). Therefore, cetyltrimethylammonium bromide (CTAB) and histidine were used as templating and assisting templating agents, respectively, to interact with the ZIF precursors, forming specific building units. Consequently, the pore size of ZIF-8 was increased to 23.1 nm and its enzyme loading efficiency was improved [87]. Similar results were also obtained using CTAB and 1,3,5-trimethylbenzene [117]. Furthermore, the nature of metal nodes and organic linkers in MOFs can affect the physical loading of the enzyme [118]. Therefore, nodes or linkers with strong affinity for the enzyme should be used to increase the enzyme uptake.

#### 3.4.2. Covalent Binding

Although immobilization by physical adsorption offers high enzymatic activity for the transesterification process, the enzymes are subject to leaching due to the weak enzyme–MOF interactions [119]. To improve enzyme stability, the weak interactions can be replaced by covalent interactions between the nucleophiles of the enzymes (free amino acids) and the organic linkers (mainly carboxylate groups) of MOFs to form peptide bonds [106]. Among the strong chemical bonds developed during enzyme immobilization, multipoint covalent attachment between the MOF and functional groups of the enzyme, such as amino, glyoxyl, and epoxy [107], leads to the formation of a rigid backbone that stabilizes the enzyme structure, enhances its resistance to unfolding and denaturation, and reduces enzyme leaching (a common feature of chemisorption) [108]. For example, *Candida antartica* lipase-B was immobilized by covalent bonding on activated isoreticular MOF-3 by dicyclohexylcarbodiimide with an enzyme loading of 0.18 mg/g, improving the enzymatic activity by up to 103 times compared to that of the free enzyme [120]. Nevertheless, in addition to the large internal surface of MOFs, other features—such as low steric hindrance and high reactive group density—are needed for effective multipoint covalent attachment. Moreover, the enzyme should retain its activity under the immobilization conditions [105].

#### 3.4.3. Cross-Linking

Enzymes can also develop intermolecular interactions with the support through covalent bonds in the presence of a multifunctional reagent that serves as a linker. There are two methods of enzyme immobilization by cross-linking: cross-linking enzyme crystal (CLEC) and cross-linking enzyme aggregate (CLEA) [121]. In CLEC, glutaraldehyde is used as the linker between the free amino groups of the enzyme and the reactive sites of a neighboring molecule. However, the addition of glutaraldehyde could seriously alter the enzyme structure, thus affecting its activity. To address this issue, inert proteins, such as gelatin or bovine serum albumin, can be added. In the case of CLEA, which is an improved version of CLEC, the introduction of a salt, nonionic polymer, or organic solvent promotes the formation of enzyme aggregates without distorting the enzyme properties. However, CLEA cannot be combined with MOFs as it does not require an external support. Taken together, immobilization by cross-linking is a simple method with a very low possibility of enzyme leaching due to the strong chemical bonds between the enzyme molecules. Furthermore, enzymes can be modified using adequate stabilizing agents to adapt to any microenvironment.

#### 3.4.4. Entrapment/Encapsulation

The entrapment of enzymes within the MOF pores requires the diffusion of the enzyme molecules through gaps that are generally smaller than the MOF cavity. Due to their high porosity, MOFs allow the adsorption of enzymes into their mesoporous structure, instead of only on their surface, thus increasing enzyme loading. Moreover, the entrapment of enzyme molecules into the MOF pores protects them from harsh denaturing conditions as the enzyme is not attached to the support and does not chemically interact with it, thus improving stability. However, enzymes immobilized by this approach exhibit mass transfer limitations and their diffusion is restricted as the substrate may not be able to access the entire active site [102]. In addition, enzyme entrapment into nano- or microporous MOFs may not be efficient, while part of the enzymatic activity may be lost due to conformational changes during diffusion into small cavities [102]. To avoid these challenges, MOFs with macroporous structure are gaining increasing attention for enzyme immobilization.

When the MOF pore sizes are smaller than the enzyme, the enzyme can only be immobilized through encapsulation into MOF crystals, a process known as co-precipitation. During this approach, enzyme immobilization simultaneously occurs with the nucleation and MOF crystal growth. Recently, mesoporous MOFs, such as MIL-100(Fe) and HKUST-1, have been tested as supports for lipase immobilization through co-precipitation [99,122]. However, low enzyme loadings were achieved due to the long-range ordering and nonuniformity of MOFs. In contrast, highly ordered MOFs with large specific areas and uniform and adjustable nano sizes could be loaded with high enzyme amounts and were effectively used for in situ enzyme encapsulation [99,109]. This technique is relatively new, and the first protein molecules directly embedded into ZIF-8 by the co-precipitation method have been reported in 2014 for cytochrome c (Cyt c) [122]. In this process, the enzymes were incubated with the precursors, i.e., zinc nitrate and 2-methylimidazole, in methanol and in the presence of polyvinylpyrrolidone (PVP) to prevent protein agglomeration in the organic solvent. Cyt c immobilized on ZIF-8 exhibited 10 times higher activity than the free enzyme due to the metal ion activation effect. Other enzymes immobilized on MOFs by the co-precipitation method are horseradish peroxidase in ZIF-8, Cyt c in ZIF-10, and lipase in ZIF-8. Enzyme encapsulation into ZIF-8 has also been achieved in an aqueous solution instead of an organic solution [123], thus eliminating the need for PVP and extending the scope of the co-precipitation method to enzymes that are significantly inactive in organic solutions. *Aspergillus niger* lipase has also been encapsulated into ZIF-8 [109], as confirmed by an amide I band observed at 1658.7 cm^−1^ in the Fourier-transform infrared spectrum, which is typical for enzymes and corresponds to the N–H bending mode. Furthermore, a biomimetic mineralization method has recently been reported as an alternative enzyme encapsulation strategy in MOFs. For instance, the encapsulation of urease using this technique affords improved thermal stability [124].

The biological functions of enzymes could be altered when they are encapsulated in MOFs, due to the interactions between them. This was investigated using catalase encapsulated in solid and hollow ZIF-8 microcrystals [125]. At a constant catalase loading, characterization of the immobilized enzyme after H_2_O_2_ degradation reaction showed no change in the structure, and kinetic study indicated no significant mass transport limitation. Nevertheless, the interfacial interactions between the enzymes and MOFs impacted their activities. To overcome this, the solid MOF microcrystals was proposed to be hollowed before enzymes encapsulation. Before the hollowing process, the enzymes were confined in the solid MOF crystals, whereas they were sealed inside of the central cavities of the hollow MOF crystals in a freestanding form, with minimum interaction. The permeable MOF shell allowed reactants to penetrate the shell and reach the enzyme, preventing the enzymes from leaching [125].

Another method that has been recently developed to overcome the interaction problem of the enzyme with the MOF the de novo approach. In this system, enzyme molecules are embedded in a MOF crystal with small pores in water mild conditions. Similar to other encapsulation techniques, the de novo approach allows MOFs with pore sizes smaller than the size of the enzymes to be used. This not only prevents leaching but also greatly expands the selection of enzymes and MOFs, making the method generally applicable for various functional applications. This concept has been used with ZIF-90 of 1 nm pore size to coat catalase molecules of 10 nm size [126]. The small pore size of ZIF-90 prevented the leaching and provided size-selective sheltering to increase tolerance against protease. The interactions between the enzyme and the MOF have marginal influence in the de novo system, which has a positive effect on enzyme activity. After being embedded in the MOF microcrystals via a de novo approach, the enzyme maintained its biological function under a wide range of conditions. By exposure to a denature reagent, urea, and high temperature of 80 °C, embedded catalase in ZIF-90 maintained its activity in the decomposition of hydrogen peroxide even, whereas free catalase was completely deactivated [127].

The immobilization of porcine pancreatic lipase (PPL) by encapsulation was explored using three different MOFs: HKUST-1, which is prepared using copper as metal nodes and BTC as the organic linker; mesoporous MIL-100(Fe); and MIL-100(Fe) containing Keggin phosphotungstic acid [122]. In particular, 5 mg of each MOF was introduced into a buffered PPL solution, followed by mild shaking at room temperature for 2 h. The encapsulation of PPL in the MOF pores was spectroscopically confirmed; the characteristic bands of MOFs in PPL@MOF were shifted relative to those of free MOFs.

Mechanochemical processes is another encapsulation method, which has been recently proposed for enhanced enzyme activity and stability. In this process, the traditional solution-based processes are replaced with a more environment-friendly mechanical alternative, such as ball milling. The process minimizes the use of organic solvents and strong acids during the MOF synthesis, allowing the encapsulation of enzymes into robust MOFs, while maintaining enzymatic biological activity. In addition, the mechanical processes are rapid and can be easily scaled-up to industrial levels. However, the advantages of this process were only demonstrated on enzyme encapsulation in ZIF-type of MOFs. The synthetic conditions required for other types, such as UiO-66-NH2 and Zn-MOF-74, were too harsh for the encapsulated enzymes to retain their activity [128].

## 4. ILs in Biodiesel Production

ILs, which are salts in the liquid form at temperatures below the boiling point of water, have recently been proposed as catalysts for biodiesel production [129,130,131,132] (Table 2). ILs are prepared from anions and cations, which can be suitably selected to adjust their properties, such as melting point, viscosity, density, water solubility, solvent selectivity, and acidity, for specific applications [93,133,134,135,136]. In addition, ILs are nonvolatile and exhibit high thermal, chemical, and electrochemical stabilities; they can efficiently dissolve a wide range of compounds, including polar, nonpolar, inorganic, and polymeric molecules. Therefore, they have been applied either as solvents or catalysts for biodiesel production [123,130,137,138,139,140]. Moreover, ILs have been used as stabilizing media for enzymes, proteins, and nucleic acids to replace organic solvents [141].

ILs can be broadly classified as simple salts and binary ILs or as ILs based on chlorometallate anions and metal-free anions [124]. Regardless of their class, ILs can be basic or acidic depending on the functional groups attached to their cations and anions [124]. To date, ILs have successfully catalyzed the transesterification and esterification of triglycerides and FFA [123,130], allowing easy separation and purification of the product, thus providing high-grade biodiesel [123,142]. In addition, ILs require a relatively low operating temperature, consequently reducing the equipment and energy costs compared to that afforded by other catalysts. Bench-scale experiments have also shown that ILs can be easily separated from the products and recycled due to their high reusability and low volatility, thus limiting the generation of waste while making the process more economical and environmentally friendly [143]. Nevertheless, ILs exhibit high viscosity, which restricts the diffusion of reactants and products [144,145].

ILs are also used as support for acid and alkali catalysts, forming heterogeneous catalysts that do not suffer from diffusion limitations and product contamination while retaining their benefits of easy recovery and reusability. For instance, using 0.1 g of Sn(pyrone)_2_ as a catalyst immobilized on 1-*n*-butyl-3-methylimidazolium tetrachloroindate (BMI·InCl_4_; 3 mL), 10 g soybean oil, and 3 g methanol, a biodiesel yield of 83% was achieved in 4 h. However, the use of Sn(pyrone)_2_ immobilized on BMI·PF_6_ under the same reaction conditions reduced the biodiesel yield to 55%. BMI·InCl_4_ was more effective than Sn(pyrone)_2_ in stabilizing the intermediates of the catalytic cycle and maintaining the catalyst phase during the separation and recharging processes [137]. However, the biodiesel yield dropped to almost zero after both immobilized catalysts were reused for three cycles due to excessive catalytic leaching. In contrast, immobilizing [Et_3_NH]Cl–AlCl_3_ and H_2_SO_4_ on 1-butyl-3-methylimidazolium bis(trifluoromethanesulphonyl) imide ([Bmim]NTf_2_) afforded 93–98.5% biodiesel production from vegetable oils, which was slightly reduced after six cycles [146].

The activity and stability of enzymes could also be increased using imidazolium ILs, because they can protect the enzyme from methanol deactivation [147,148]. For example, using 1-butyl-3-methylimidazolium trifluoromethanesulfonate ([Bmim][TfO]) with Novozym^®^435, the biodiesel yield increased eight folds compared to that afforded by the solvent-free system [149], while the application of 1-butyl-3-methylimidazolium hexafluorophosphate ([Bmim][PF_6_]) with the same enzyme resulted in 60% biodiesel yield, which was higher than that achieved using *n*-hexane under the same conditions (40%) [149]. In addition, a yield of 60% was achieved in 4 h using the hydrophobic IL [Bmim][PF_6_] at 45 °C with a methanol/oil ratio of 5:1 and 30% enzyme loading, whereas yields of up to 15% were obtained using hydrophilic ILs under the same conditions [150]. The reusability of the [Bmim][PF_6_]–lipase system was also studied, showing a 13.3% decrease in enzyme activity from the first to the fourth cycle (120 min each) [151]. To further enhance the stability of the IL–enzyme system, silica xerogel was used as a support to immobilize *Burkholderia cepacia* lipase through covalent binding, while the sol–gel method was combined with a protic IL during synthesis to improve the system’s morphological and physicochemical characteristics [152].

### 4.1. Acidic ILs

#### 4.1.1. Acidic ILs as Catalyst Supports

The high thermal stability of acidic ILs coupled with the promoted release of H^+^ ions at high temperatures contributed to improving their activity [159]. Therefore, the Brønsted acidic IL 1-butyl-3-methylimidazoliumtosylate ([Bmim][CH_3_SO_3_]) was used to enhance the FeCl_3_ activity in biodiesel production from untreated jatropha oil [130]. Using the IL–FeCl_3_ system, biodiesel was obtained in 99.7% yield after 6 h at 120 °C, while the yield decreased to 12% using only [Bmim][CH_3_SO_3_], because the metal ions supplied the Lewis acidic sites. Moreover, trivalent metal ions provide more sites than bivalent ions.

The catalytic activity has also been found to increase with the atomic radius of metal ions [2]. When tetrabutylammonium iodide (TBAI) was used as a phase transfer agent, the catalytic activity was improved due to the inherent lipophilic nature of the IL and its ability to provide methoxide anions in the oil phase [160,161]. High biodiesel yields were also achieved under mild operation conditions using the TBAI–ZnO system in different oil sources, including lard, fish, waste cooking oil, linseed, soybean, and jatropha. A maximum yield of 96% was obtained from soybean oil after 7 h at 65 °C. However, the high solubility of TBAI–ZnO did not favor its recovery and reuse, in contrast to [Bmim][CH_3_SO_3_]–FeCl_3_, which could be easily separated and reused.

#### 4.1.2. Acidic ILs as Sole Catalysts

ILs have also been considered as alternative catalysts for the transesterification of triglycerides or the esterification of FFA to produce biodiesel [162]. No product was detected when a mixture of oleic acid and ethanol was stirred at 78 °C in the absence of catalysts, suggesting that the catalyst plays a significant role in the reaction progression. When sulfuric acid was added at 70 °C, 61% conversion was achieved in 6 h. In contrast, monocationic acidic ILs, such as *N*,*N*,*N*-trimethyl-*N*-propanesulfonic acid ammonium hydrogen sulfate [TMPSA][HSO_4_], 3-methyl-1-(3-sulfopropyl)-imidazolium hydrogen sulfate [MIMPS][HSO_4_], and 1-sulfopropylpyridinium hydrogen sulfate [PyPS][H_2_SO_4_], increased the conversion to 85–87% [162]. Moreover, the dicationic IL *N*’,*N*’,*N*’,*N*’-tetramethyl-*N*,*N*’-dipropanesulfonic acid ethylene diammonium hydrogen sulfate ([TMEDAPS][HSO_4_]) increased the esterification conversion to 95% due to its higher Brønsted acidity than that of the other ILs. Moreover, all ILs exhibited high catalytic stability with a conversion drop of less than 3% after six reuse cycles.

To confirm the effect of the IL acidic strength on biodiesel production, the potential of 1-methylimidazolium hydrogen sulfate ([Hmim][HSO_4_]), 1-*n*-butyl-3-methylimidazolium methylsulfate ([Bmim][MeSO_4_]), 1-butyl-3-methylimidazolium hydrogen sulfate ([Bmim][HSO_4_]), [Bmim][CH_3_SO_3_], and tributylmethylammoniummethylsulfate ([TBMA][MeSO_4_]) for the esterification of oleic acid was investigated [153]. The acidity of the IL anions increased in the order [CH_3_SO_3_]^2−^ < [HSO_4_]^2−^ < [MeSO_4_]^3−^, whereas the strength of the cations followed the order [TBMA]^+^ < [Bmim]^+^ < [Hmim]^+^. Among them, [Hmim][HSO_4_] exhibited the best catalytic activity, as it offered two acidic sites due to its high anionic and cationic strength. The conversion of oleic acid after 6 h at 90 °C in the presence of 10 wt % [Hmim][HSO_4_] and a molar ratio of 10:1 reached 88.5%, which was five and six times higher than the conversions achieved with [Bmim][CH_3_SO_3_] and [TBMA][MeSO_4_], respectively. By increasing the [Hmim][HSO_4_] loading to 15 wt % and the methanol/acid ratio to 15:1, the conversion of oleic acid increased to 95% after 8 h at 110 °C. A slight reduction in enzyme loading to 14 wt % reduced the biodiesel yield to 90% under the same operating conditions [153].

Despite the good activity of heteropoly acid-based ILs, their strong polarity makes them highly soluble in the alcohol used for biodiesel production. Therefore, water and methanol must be completely evaporated to separate and reuse them, increasing the energy and economic requirements. Thus, similarly to soluble enzymes, heterogeneous ILs—either polymeric or immobilized on a suitable support—have been proposed to simplify their separation from the reaction mixture.

#### 4.1.3. Polymeric Acidic ILs

Polymeric acidic ILs have been extensively studied as they combine the desired catalytic properties of ILs and the insolubility of polymers in commonly used organic solvents, facilitating their recovery and reuse [163]. Due to their heterogeneity, polymeric ILs (PILs) have small specific surface areas, thus preventing the access of sterically hindered reactants to their catalytic sites. To produce a uniform pore structure in PILs, a suitable hard nanoparticle-based template can be used during the polymerization process, which can be easily removed after the polymer formation. For example, a porous PIL catalyst was successfully prepared using Fe_3_O_4_ nanoparticles. Prior to polymerization with 1-vinyl-3-(3-sulfopropyl)imidazolium hydrogen sulfate [VSIM][HSO_4_], the Fe_3_O_4_ nanoparticles were modified with 3-methacryloxypropyltrimethoxysilane. At the end of the process, the nanoparticles were removed by a water/ethanol/hydrogen chloride solution and ultrasonication, affording the acidic microporous catalyst [VSIM][HSO_4_], which was applied in biodiesel production from oleic acid. A maximum yield of 92.6% was achieved after 4.5 h at 80 °C with a methanol/acid ratio of 12:1 and a catalyst loading of 5 wt %. Further application of the PIL in the catalytic conversion of oleic acid and caper spurge oil to biodiesel at 75 °C and a methanol/oil ratio of 17:1 afforded 95.3% and 97.1% conversions, respectively, in 3 h [157]. In addition, after five reuse cycles of [VSIM][HSO_4_], the caper spurge oil conversion decreased by only 4% due to the hydrophobic regulatory effect of the catalyst, which ensured the maintenance of the activity [156].

#### 4.1.4. Immobilized Acidic ILs

To further improve the separation and reusability of acidic ILs, an acidic IL- functionalized mesoporous melamine–formaldehyde polymer (MMFP–IL) was prepared to catalyze the transesterification of oleic acid with methanol [164]. Using a methanol/acid ratio of 12:1 and a catalyst loading of 4 wt % at 90 °C, a biodiesel yield of 95% was achieved in 3 h. The strong covalent bond interaction between MMFP and IL enhanced the stability of the catalyst and allowed its repeated use with only 7% yield reduction after the fourth cycle. In another study, biodiesel was produced in 98.5% yield through the esterification of oleic acid after 4 h at 100 °C in the presence of the heteropoly acid (SiW_12_O_40_)-based IL (SWIL), which was applied either directly or supported on silica [158]. Although SWIL exhibited high stability and reusability, its direct application required complete evaporation of water and methanol due to its high solubility. In contrast, simple filtration could be used to recover SWIL supported on solid silica. While unsupported SWIL exhibited high stability over several reuse cycles, the activity of SWIL/SiO_2_-1 and SWIL/SiO_2_-2 with different silica weight ratios decreased by 26.85% and 57.59%, respectively, after the seventh reuse cycle. This significant decrease was attributed to the surface overloading with SWIL, reduction in specific surface area and pore volume, and high mass transport limitations.

### 4.2. Basic ILs

Although significant progress has been made in the application of Brønsted acidic ILs in esterification and transesterification reactions for biodiesel production, reports on Brønsted basic ILs are relatively scarce, with most studies focusing on their role as solvents and catalysts in organic reactions [165]. However, a maximum biodiesel yield of 98.5% was achieved after 4 h at 55 °C using a basic IL with a catalyst loading of 0.4% and a methanol/cottonseed oil ratio of 12:1. The conversion decreased to only 96.2% after seven reuse cycles (Table 3) [123].

### 4.3. Limitations of ILs

Although ILs have shown promising results for biodiesel separation and glycerol removal, the effect of the accumulated byproducts on biodiesel recovery has not been extensively studied. In addition, the acidity of ILs when used as catalysts remains unknown, as it cannot be determined by a specific method. Although ILs are very stable and can be repeatedly used without losing their activity, water and methanol need to be completely evaporated after each cycle so that the pure ILs can be recovered. Heterogeneous PILs can simplify the separation process, but the removal of the product by solvent extraction may reduce the IL catalytic strength in subsequent runs. Alternatively, ILs can be immobilized on porous structures such as solid silica. However, this strategy reduces the specific surface area and pore volume and increases the mass transport limitations during the repeated use of immobilized ILs, resulting in significant activity reduction [143]. Therefore, similar to enzymatic processes, MOFs have also been suggested to immobilize ILs.

## 5. Use of Immobilized Enzyme in Biodiesel Production

Lipase is attached in commercially available Novozym@435 by cross-linking divinylbenzene and methacrylic acid on polyacrylic resin. These cross-liking agents have high protein affinity, which reduce enzyme leaching, while minimizing the negative effect of chemisorption on enzyme activity. Functional groups carried by the monomers of a cross-linking polymer can be selected according to the immobilized enzymes. These functional groups can facilitate the binding of the enzyme to the support or increase the affinity of the substrate and the immobilized enzyme, thereby increasing the enzyme activity [169]. Therefore, Novozym@435 was shown to exhibit high efficiency for biodiesel production [170]. It was successfully used with waste cooking oil achieving a conversion up to 93%, with high stability [171].

The replacement of conventional organic solvents with greener ILs has opened up new opportunities for Novozyme@435 in biodiesel production. The use of ILs containing long alkyl chains on the cation has the important advantage of producing homogeneous systems at the start of the reaction but, when the reaction is complete, a three-phase system is created that allows selective extraction of the products using straightforward separation techniques, while the IL and the enzyme can be reused [149]. Biodiesel yield from soybean oil using Novozym@435 in [Emim][TfO] IL was 80% after 6 h, which was eight times higher than that archived in solvent-free system and 15% higher than the that using tert-butanol as an additive [172] at the same conditions. Other ILs that showed promising results with Novozyme@435 are [C16MIM] [NTf2] and [BMIm][PF6], achieving 98% and 86% biodiesel yields from Triolein and microalgae oil, respectively [149,150].

As mentioned earlier, MOFs have been used as promising carriers for the enzyme immobilization. The MOF-enzymes biocomposites exhibited excellent biocatalytic properties, improved stability, and reusability. By using lipase of the same genus, *Candida* sp., of that used in Novozym@435, encapsulated inside ZIF-67, 78% biodiesel yield was achieved [173]. By using *Rhizomucor miehei* lipase encapsulated in X-shaped ZIF-8, a biodiesel production conversion of soybean oil reached 92.3% after 24 h reaction time. The enzyme retained 84.7% of its initial activity after 10 repeated cycles [15]. By using hierarchical mesoporous (ZIF-8) to immobilize *Burkholderia cepacia* lipase (BCL) into surface adsorption the conversion of transesterfiction reaction 93.4% yield, when the optimum conditions for biodiesel production were transesterification time 12 h with three-step addition of alcohol at 4 h intervals and reaction temperature 40 °C. There was no significant drop in conversion yield relative to original activity for BCL-ZIF-8 when continuously reused for eight cycles [174].

On the other hand, when Novozym 435 used in esterification of free fatty acids from palm oil fatty acid distillate (PFAD), 93% conversion was obtained after 2.5 h using ethanol with 1.0 wt % of Novozym 435 at 60 °C. Novozym 435 was reused 10 times with conversion reaching 88% and 65% after the 11th reaction with ethanol and methanol, respectively [175].

When Novozym 435 was used in esterification reaction in the presence IL [BMIM][PF_6_] and Methyl acetate as the acyl acceptor, a biodiesel yield of 80% was achieved at the optimum conditions of 14:1 oil:acyl acceptor molar ratio; 20% (*w* immobilised lipase/*w* of oil) and a temperature in the range of 48–55 °C. After nine repeated runs, a decline in in lipase activity was observed after the sixth run [176]. 

By immobilizing lipase from *Candida rugosa* in magnetic Fe_3_O_4_@MIL-100(Fe) MOF, prepared by coating Fe_3_O_4_ magnetite with porous MIL-100(Fe) MOFusing amide linkages, a maximum biodiesel conversion of 92.3% was obtained at a methanol/oil molar ratio of 4:1, with a three-step methanol addition manner, and a reaction temperature of 40 °C. The biocatalyst was recycled easily by magnetic separation without significant mass loss, and displayed 83.6% of its initial activity after five runs, thus allowing its potential application for the cleaner production of biodiesel [177].

A covalent immobilized *Candida antarctica* lipase (CALB) onto the bio-based MOF with adenine as the organic ligand based on the concept of biomimetic assembly was used in the esterification of oleic acid with methanol for biodiesel production. The highest yield of 98.9% was obtained under the optimized conditions: methanol/oil ratio of 3.65:1, a reaction temperature of 46.3 °C, a CALB@MOF loading of 117.77 mg and a reaction time of 11.55 h [178]. Table 4 shows a summary of comparison between the performance of lipase immobilized on different supports in biodiesel production.

## 6. IL–MOF Systems for Biodiesel Production

ILs have been used in biodiesel production as catalysts or enzyme stabilization solvents for the transesterification reaction and as solvents for lipid extraction from oil bio-sources. However, their commercial application is hindered by several drawbacks, such as their high cost, high viscosity, complex recovery, and large concentration needs when used as catalysts. Although most of the drawbacks can be outweighed by their overall benefits, their high viscosity and separation complexities remain serious challenges. Therefore, ILs have been heterogenized using solid materials to form supported ILs (SILs), which can be more easily separated and recovered while minimizing diffusion limitation, a major drawback of heterogeneous catalysts [156]. Since SILs combine the advantages of homogeneous and heterogeneous catalysts, they provide new opportunities for catalytic reactions.

MOFs have recently been suggested as good support for ILs (Table 5), as their highly porous crystalline nature can overcome the mass transfer limitation problems encountered with conventional supports, such as silica [179,180]. ILs immobilized on MOFs also exhibit excellent activity and easy recovery compared to their non-immobilized counterparts [180]. For instance, an acidic catalyst prepared by immobilizing [SO_3_H-(CH_2_)_3_-HIM]_3_PW_12_O_40_ on MIL-100 converted oleic acid to biodiesel in 94.6% yield after 5 h at 111 °C with an acid/ethanol ratio of 1:11. The increased catalytic activity was also attributed to the additional Brønsted acid resulting from the sulfonic group in the IL and the strong interaction between the Lewis and Brønsted acids. Nevertheless, repeated use of the MOF-impregnated IL increased leaching and reduced its activity. In contrast, the encapsulation of IL into MIL-100 reduced the oleic acid conversion by only 5% with no obvious leaching after six cycles [44].

The benefits of encapsulation into MOFs was further demonstrated by encapsulating di-cationic acidic ILs (DAILs; 1,4-bis[3-(propyl-3-sulfonate) imidazolium] butane hydrogen sulfate) into MIL-100(Fe), which was used for the esterification of oleic acid with methanol [18]. A conversion of 93.5% was achieved after 5 h at 67 °C with a catalyst amount of 15% and a methanol/oil ratio of 8:1. The reusability of the catalyst was also tested, showing a 7.5% reduction in activity after five cycles, which was significantly lower compared to the 68% reduction observed with the MOF-impregnated IL. This significant activity reduction was attributed to the increased IL leaching. However, MOF-encapsulated ILs were not susceptible to leaching and DAILs were effectively encapsulated into the MIL-100(Fe) MOF cages, thus exhibiting significantly higher catalytic activity. Only a slight reduction in activity was observed due to MOF blockage.

The importance of IL encapsulation into MOFs to overcome the leaching problem was also confirmed by the encapsulation of 2-mercaptobenzimidazole into MIL-101(Cr) [18], which was used to catalyze the esterification of oleic acid with methanol [115,184]. A biodiesel yield of 91% was achieved after 4 h at 67 °C with an acid/methanol molar ratio of 1:10 and a catalyst loading of 11 wt %. The conversion was reduced by only 8.9% after six cycles due to the blockage of the sites during recovery. However, when the IL was impregnated on the MOF, the yield decreased by 71.6% due to IL leaching from the MOF.

Similar results were also observed when [(CH_2_COOH)_2_IM]HSO_4_ was encapsulated into H-UiO-66. The catalyst was used for the esterification of oleic acid with methanol to produce biodiesel. The optimum yield was 93.8% after 5 h at 80 °C at a methanol/acid ratio of 10.39:1 and a catalyst loading of 6.28 wt %. However, separately using [(CH_2_COOH)_2_IM] HSO_4_ and H-UiO-66 reduced the yield from 93.82% to 90.95% after five uses, indicating the important synergistic effect between the IL and MOF, which was attributed to the Lewis acid sites of the unsaturated Zr atoms of the MOF and the Brønsted acid sites of the IL. In addition, the catalyst showed an activity loss of less than 3% after five reuse cycles, and its separation was simple due to the strong interaction between the IL and MOFs [115,181].

Functionalized MOFs were also prepared and used as supports for ILs, limiting the restrictions of the preparation conditions on the catalyst while reducing the leaching problem encountered with impregnated ILs. In particular, functionalization with Keggin-type polyoxometalate (POM) was suggested to enhance the attachment of ILs. A phosphomolybdenum-based sulfonated IL impregnated on POM-functionalized MIL-100(Fe) was used for the transesterification of soybean oil. The strong interaction between the Lewis and Brønsted acids ensured high surface acidities, which improved the catalytic activity. Using a methanol/oil ratio of 30:1 and a catalyst loading of 9 wt % at 120 °C, a biodiesel yield of 92.3% was achieved in 8 h. After five cycles, the catalytic activity was reduced to 89.5%, whereas the activity dropped to 18.2% in the absence of POM functionalization [185]. Similar results were also obtained by incorporating sulfonated acidic ILs into POM-functionalized UiO-66-2COOH. The resulting catalyst possessed high surface area and was used for biodiesel production from oil-based substrates with high FFA (9%) and water (3%) content through the transesterification of triglycerides and the esterification of FFA in one step [180]. The biodiesel yield after 6 h with a catalyst loading of 10 wt % and a methanol/oil ratio of 35:1 at 110 °C was 95.8%. In addition to its high activity, the catalyst exhibited good stability and was reused five times with insignificant activity loss. Thus, this catalyst was presented as a good choice for biodiesel production from low-cost feedstock oils with relatively high FFA and water content.

To enhance the separation of IL@MOF catalysts, an acidic robust catalyst was prepared by encapsulating imidazolium dihydrogen sulfate on MOF [Fe_3_O_4_@NH_2_-MIL-88B(Fe)] and used to produce biodiesel from oleic acid esterification with ethanol [182]. A maximum yield of 93.2% was achieved after 4 h at 90 °C with an ethanol/oil ratio of 10.5:1 and a catalyst dosage of 8.5 wt %. Due to the magnetic metal Fe, the catalyst was easily recovered from the reaction mixture without any loss using an external magnetic field. The stability of the recovered catalyst was tested; its activity was maintained for six cycles under normal pressure. Good results were also obtained using magnetic MOFs for the preparation of hybrid alkaline catalysts. For example, a novel catalyst was recently prepared by immobilizing a core–shell amino-functionalized basic IL catalyst on magnetic structured Fe_3_O_4_@HKUST-1 by a post-synthetic modification technique using different solvents such as water, ethanol, and *N*,*N*-dimethylformamide. The obtained catalyst was tested for the transesterification of soybean for biodiesel production using a methanol/oil molar ratio of 30:1 and a catalyst dosage of 1.2 wt % at 65 °C [183]. After 3 h, a biodiesel yield of 92.3% was achieved. Furthermore, the presence of the magnetic metal allowed easy recovery of the catalyst because of the application of an external magnetic field, and the catalytic activity remained almost unchanged after five reuse cycles.

## 7. Cost Analysis and Viability of Immobilized Enzymes

As mentioned earlier, the advantages of enzymatic approach over conventional alkali-based processes have been clearly demonstrated in biodiesel production from non-refined feedstock. The enzymatic approach is less energy intensive, more environment friendly, simplifies the separation of the byproduct glycerol, and eliminates the need for water-washing step that consequently reduce wastewater treatment cost [186,187]. Most importantly however, enzymes are insensitive towards free fatty acids (FFA) content in the feed, allowing it to be used with low quality feedstock. They even catalyze the FFA together with the transesterification of the triglycerides, which increases the overall biodiesel yield. Nevertheless, the high cost of enzymes remains the main challenge facing the commercialization of enzymatic biodiesel production processes [187,188]. In an economic study on the production of 1000 tons of biodiesel from palm oil, alkali process was found to be more feasible that enzymatic process, when the enzyme was used in a soluble form [189]. It is obvious therefore that enzymatic process can only be feasible if the enzymes are repeatedly used with maintained activity. As mentioned before, this is achieved by enzyme immobilization on a suitable support. By using immobilized enzyme in the economic [189], the feasibility of the process increased. Although the alkali catalyzed process was found to be still more feasible, the study limited the number of reuses to only five. If reusability is increased, the lipase process becomes more feasible. Allowing the use of low-quality feedstock also favors the use of enzymes over alkaline processes. In 2006, a biodiesel production line of 20,000 t/y capacity was built in China using waste cooking oil as feedstock, in which a combination of different immobilized lipases has been used as a catalyst [123,190].

The successful immobilization of the enzyme on the support for maintained activity and stability, with minimization of mass transfer limitation, play major role in shifting the economic balanced towards the enzymatic process. Although many technologies have been developed for lipase immobilization at lab scale, only a few are industrialized. The main challenges are the high cost of the carrier support, low enzyme capacity and enzymatic activity and stability retaining challenges. The most widely used immobilized enzyme in biodiesel production is Novozym@435, which is sold at a price of about US$1000/kg [188]. In addition, the advancement in biotechnology promises to offer new enzymes of lower production costs and higher catalytic activity and stability, which would further improve the feasibility of enzymatic process. It has been recently reported that immobilized lipase products, specifically designed for biodiesel production, have been developed with a reduced price of about $150/kg [116]. This will pave the way for commercial use of enzymatic processes.

Another aspect that needs to be considered in the environmental factor, which may not be readily transferred into cost. Enzymes are more environment friendly process, which reduces the wastewater production. In that regard, the use of green solvents, instead of conventional organic solvents that require additional separation and purification units are therefore essential. Among the most promising alternative solvents are ILs, which as mentioned earlier can further enhance enzyme reusability with enhanced mass transfer [123]. However, ILs are generally more expensive than organic solvents. The cost of the most commonly used ILs in enzymatic biodiesel production, namely 1-*n*-butyl-3-methylimidazolium tetrafluoroborate ([BMI][BF_4_]) and 1-*n*-butyl-3-methylimidazolium hexafluorophosphate ([BMI][PF_6_]), are about 25 times more than organic solvent. Therefore, feasible use of enzyme-IL systems requires repeated reuse with maintained activity and stability and efficient separation of the products is another important factor [190]. Additionally, deep eutectic solvents (DESs), is a potential replacement to ILs, which are more cost-effective and environmentally friendly. The properties of DESs can be finely tuned, similar to those of ILs, by selecting different cation and anion combinations. They have characteristics similar to those of ILs, such as high purity, ease of preparation, non-toxicity, biodegradability, requirement of mild reaction conditions, and insensitivity to water [191]. In addition, BASF has recently commercialized four MOF materials, including BASOLITE-A100 (MIL-53), BASOLITE-C300 (HKUST-1), and BASOLITE-Z1200 (ZIF-8) with prices ranging between 10 to 15 US$/g, making these MOFS only affordable for research purpose at this time. However, with advance in raw materials selection and synthesis technology, lower prices that are comparable to that of synthetic zeolites may be achieved for large scale synthesis of some MOFs in the future [192].

## 8. Conclusions

In this review, we summarized the recent advances in catalytic transesterification for biodiesel production. The use of MOFs as immobilization support materials for enzymes or ILs enhanced the reaction yield without increasing operating costs. Moreover, this strategy did not affect the stability of the catalysts, and their reusability was improved and the mass transfer limitations within the immobilization matrix were restricted. Therefore, the application of MOFs can significantly reduce the energy and economic requirements for the production process, making biodiesel a competitive alternative for conventional fuels. Further comparison of different lipase immobilization techniques on MOFs showed that the encapsulation of enzymes in a support framework instead of their post-preparation adsorption improves the catalyst stability due to reduced leaching. Although supporting homogeneous chemical catalysts or enzymes inside ILs can be used to form heterogeneous catalysts while avoiding diffusion limitations, the separation of the product by solvent extraction reduced the catalytic activity. This further indicates the superior properties of MOFs as immobilization supports compared to other materials.

## Figures and Tables

**Table 1:** Examples of MOFs used in biodiesel production.

MOF	Oil Source/Alcohol	Synthesis	Pore Size	Time	Temperature/Energy	Yield	Ref.
CuBTC-MOF	Palm oil/methanol (1:5)	Solvothermal method	1.68 cm^3^/g	4 h	60 °C	91%	[100]
ZrSiW/Fe-BTC	Oleic acid/methanol (20:1)	Hydrothermal method	0.135 m^3^/g 191.5 m^2^/g	4 h	160 °C	85%	[102]
ZrSiW/UiO-66	Oleic acid/methanol (20:1)	Hydrothermal method	0.243 m^3^/g 249.4 m^2^/g	4 h	150 °C	98%	[102]
Mg_3_(bdc)_3_(H_2_O)_2_	Oleic acid/methanol (15:1)	Microwave irradiation	-	8 min	150 Watt	97%	[103]
MIL-53 (Fe)	Oleic acid/ethanol (1:16)	Ultrasonic irradiation	239 cm^3^/g 1050 m^2^/g	15 min	150 Watt	96%	[104]
MIL-53 (Fe)	Oleic acid/*n*-butanol (1:16)	Ultrasonic irradiation	239 cm^3^/g 1050 m^2^/g	15 min	150 Watt	98%	[104]
HKUST-1	Palm oil/ethanol (1:1)	Electrolysis	324.33 m²/g 0.19 cc/g	2 h	room temperature, 15 V	54%	[105]

**Table 2:** Examples of ILs used in biodiesel production.

Catalyst	Substrate	Ionic Liquid	Loading (*w*/*w*%)	Temperature (°C)	Alcohol Ratio	Time (h)	Yield (%)	Reusability	Ref.
Catalytic assist Acidic IL—Transesterification									
KOH	Jatropha oil	[Bmim][CH_3_SO_3_]–FeCl_3_	13.8	120	6:1	5	99.7	Not reported	[143]
No catalyst			-				12	Not reported	[143]
Acidic IL—Esterification									
No catalyst	Oleic acid	[HMIM][HSO_4_]	15	110	15:1	8	95.9	Not reported	[153]
Acidic IL—Transesterification									
No catalyst	Waste cooking oil	[TBP][NTf_2_]	4.5	70	12:1	1	29.7	4 cycles with 4% decrease in activity	[154]
				60	18:1	10	81.0	Not reported	[154]
	Palm oil	[HSO_3_-Bmim][HSO_4_]	9.2	108	11:1	8	92.9	Not studied the conventional experiment	[155]
Microwave (168 W)	Palm oil	[HSO_3_-Bmim][HSO_4_]	9.2	108	11:1	6.4	98.9	6 cycles with 14.1% decrease in activity	[155]
Microwave (120 W)		[MIM][HSO_4_]	10	Not mentioned	12:1	6	4.0	Not reported	[155]
Polymeric IL—Esterification									
No catalyst	Oleic acid	[VSIM][HSO_4_]	8.5	80	12:1	4.5	92.6	6 cycles without leaching acidic sites	[156]
		FnmS-PIL (1a, C8)	5	75	17:1	3	95.3	Not reported	[157]
Polymeric IL—Transesterification									
No catalyst	Caper spurge oil	FnmS-PIL (1a, C8)	5	75	17:1	3	97.1	5 cycles with 4% decrease in activity	[157]
Immobilized IL—Esterification									
No catalyst	Oleic acid	SiW_12_O_40_ (SWIL/SiO_2_)	4	100	30:1	4	98.5	Significant reduction after 7 cycles	[158]

**Table 3:** Comparison of different types of basic ILs.

Catalyst	Substrate	Ionic Liquid	Loading (*w*/*w*%)	Temp (°C)	Alcohol Ratio	Time (h)	Yield (%)	Reusability (Cycles)	Ref.
No catalyst	Cottonseed oil	Bis-(3-methyl-1-imidazolium)-ethylene dihydroxide	0.4	55	12:1	4	98.5	7	[123]
Support									
SBA-15 silica	Soybean oil	4-Butyl-1,2,4-triazolium hydroxide on SBA-15	7.0	Not reported	20:1	4	95.4	4	[166]
Boehmite nanoparticles (BNPs)		Chlorocholine hydroxide (CCH) on BNPs	4.1	60	11:1	4.4	95.2	5	[167]
No catalyst		1-Butyl-3-methyl morpholine hydroxide ([Hnmm]OH)	4.0	70	8:1	1.5	97.0	5	[168]

**Table 4:** Performance of lipase immobilized on different supports in biodiesel production.

Enzyme	Support	Immobilization Support	Temperature (°C)	Time (h)	Biodiesel Yield%	Ref.
Novozym@435	-	Cross linking	60	2.5	93	[175]
Novozym@435	[BMIM][PF_6_]	Cross linking	55	6	80	[176]
*Candida rugosa*	Fe_3_O_4_@MIL-100(Fe)	Covalent attached	40	60	92	[177]
*Candida antarctica*	CALB@MOF Bio-based	Encapsulated	46	12	99	[178]
Novozym@435	[Emim][TfO]	Cross linking	50	12	80	[172]
Novozyme@435	[C16MIM] [NTf2]	Cross linking	60	6	98	[149]
Novozyme@435	[BMIm][PF6]	Cross linking	40	48	86	[150]
*Candida sp*	Zif-67	Encapsulation	45	60	78	[173]
*Rhizomucor miehei*	X-shaped ZIF-8	Encapsulation	45	24	92	[15]
*Burkholderia cepacia*	Mesoporous ZIF-8	Adsorption	40	12	93	[174]

**Table 5:** IL–MOF systems used in biodiesel production.

MOF	Substrate	Ionic Liquid	Loading (*w*/*w*%)	Temp (°C)	Alcohol Ratio	Time (h)	Yield (%)	Reusability	Ref.
Encapsulation—Esterification									
MIL-100	Oleic acid	[SO_3_H-(CH_2_)_3_-HIM]_3_PW_12_O_40_	15	111	11:1	5	94.6	6 cycles	[180]
MIL-100(Fe)		(DAILs, 1,4-bis[3-(propyl-3-sulfonate) imidazolium] butane hydrogen sulfate)	15	67	8:1	5	93.5	5 cycles	[18]
MIL-101(Cr)		2 -Mercaptobenzimidazole with electron rich-SH groups (MBIAILs)	11	67	10:1	4	91.0	6 cycles	[18]
H-UiO-66		[(CH_2_COOH)_2_IM]HSO_4_	6.28	80	10:1	5	93.8	5 cycles	[181]
(Fe_3_O_4_@NH_2_-MIL-88B(Fe))		1,4-Butanediyl-3,3′-bis-(3-sulfopropyl) imidazolium dihydrogensulfate	8.5	90	10.5:1	4	93.2	6 cycles Insignificant reduction	[182]
Encapsulation—Transesterification									
Fe_3_O_4_@HKUST-1	Soybean oil	Fe_3_O_4_@HKUST-1	1.2	65	30:1	3	92.3	5 cycles Insignificant reduction	[183]
Functionalized with POM—Transesterification									
MIL-100(Fe)	Soybean oil	Phosphomolybdenum-based sulfonated	9	120	30:1	8	92.3	5 cycles with 89.5% biodiesel yield	[44]
UiO-66-2COOH	Oil-based with high FFA (9%) and water (3%) content	polyoxometalate-based sulfonated	10	110	35:1	6	95.8	5 cycles Insignificant reduction	[180]
